# Accurate genomic predictions for chronic wasting disease in North American elk

**DOI:** 10.1093/g3journal/jkag082

**Published:** 2026-03-31

**Authors:** Christopher M Seabury, Eric K Bhattarai, Marcel Brun, Charles D Johnson, Nicholas J Haley, Gordon B Mitchell, Tracy A Nichols

**Affiliations:** Department of Veterinary Pathobiology, Texas A&M University, College Station, TX 77843, United States; Department of Veterinary Pathobiology, Texas A&M University, College Station, TX 77843, United States; Genomics Core, Texas A&M AgriLife Research, College Station, TX 77845, United States; Genomics Core, Texas A&M AgriLife Research, College Station, TX 77845, United States; Department of Microbiology and Immunology, Midwestern University, Glendale, AZ 85308, United States; Canadian Food Inspection Agency, Ottawa Laboratory-Fallowfield, 3851 Fallowfield Road, Ottawa, Ontario K2J 4S1, Canada; USDA-APHIS-VS-Cervid Health Program, Fort Collins, CO 80526, United States

**Keywords:** chronic wasting disease, genome-wide association, genomic prediction, heritability, elk

## Abstract

The geographic expansion of chronic wasting disease (CWD) in North American elk (*Cervus canadensis*) has not been well mitigated by traditional best management practices, diagnostic surveillance, and depopulation of positive herds. Using a custom Affymetrix Axiom genetic variant array, we demonstrate that differential susceptibility to CWD is highly heritable $\left(h^{2}=0.630\pm 0.061-0.678\pm 0.056\right)$ among farmed North American elk, with loci other than *PRNP* involved. Genome-wide association analyses using 173,674 quality filtered variants for a geographically diverse cohort of 904 farmed North American elk (*n* = 357 CWD positive; *n* = 547 CWD nondetect) confirmed the prion gene (*PRNP* codon 132 Met→Leu and promoter variants) as a large-effect risk locus (*P* < 5.135E-08), as evidenced by the estimated proportion of phenotypic variance explained (PVE ≥ 0.032). However, more phenotypic variance was collectively explained by loci other than *PRNP*. Genomic best linear unbiased prediction (GBLUP; *n* = 173,674 markers) with *k*-fold cross-validation (*k* = 3; *k* = 5) and random sampling (*n* = 50 iterations) for the same cohort of 904 farmed North American elk produced mean genomic prediction accuracies ≥ 0.791, thereby providing a foundation to explore a genomically estimated CWD genetic improvement program.

## Introduction

Chronic wasting disease (CWD) was first observed among captive mule deer (*Odocoileus hemionus*) and black-tailed deer (*Odocoileus hemionus columbianus*) residing in several Colorado wildlife facilities during the late 1960s, with subsequent histological recognition as a prion disease by the late 1970s (Williams and Young 1980; Moreno and Telling 2018 ). Following these initial observations, CWD was detected and further described in free-ranging North American (NA) mule deer, elk (*Cervus elaphus nelsoni*), white-tailed deer (*Odocoileus virginianus*), and moose (*Alces alces shirasi*), with a panoptic geographic expansion of the disease predicted by ongoing diagnostic surveillance of farmed and free-ranging populations of these species (Moreno and Telling 2018; Gavin et al. 2019; Osterholm et al. 2019). To date, CWD has been detected within at least 36 US states and 5 Canadian provinces (Moreno and Telling 2018; Gavin et al. 2019; Osterholm et al. 2019; Richards 2025). Relevant to the global distribution of CWD, countries such as Norway, Finland, South Korea, and Sweden have also detected the disease in free-ranging reindeer (*Rangifer tarandus*; Norway), red deer (*Cervus elaphus*; Norway), moose (Norway, Finland, Sweden), farmed elk (i.e. initially via importation; South Korea), farmed sika (*Cervus nippon*; South Korea), and farmed red deer (South Korea) (Moreno and Telling 2018; Gavin et al. 2019; Osterholm et al. 2019; Ågren et al. 2021; Park et al. 2025). Notably, recent studies hypothesize that 1 or more transmissible forms of CWD may have emerged from sporadic CWD in Norwegian reindeer, moose, and perhaps red deer (Mysterud et al. 2020; Güere et al. 2021), thereby underscoring the inability to suppress the continued expansion of this disease, especially among wild populations. Moreover, historic best management practices, including containment and depopulation of CWD-positive herds, has not effectively prevented the emergence of CWD in new geographic locations (Moreno and Telling 2018; Gavin et al. 2019; Osterholm et al. 2019; Ågren et al. 2021). However, when modern best management practices and genomic selection were implemented within a US CWD-positive white-tailed deer farm (i.e. as a demonstration project), the prevalence of CWD remained zero for more than 4 years, including up to and beyond the conclusion of the present study (Seabury 2023). Importantly, previous genome-wide association studies (GWAAs) with marker-based heritability estimates and genomic prediction with cross-validation have directly enabled novel CWD management strategies in farmed white-tailed deer (Seabury et al. 2020, 2022; Seabury 2023; Nichols 2024). No similar studies have been reported for farmed NA elk. Therefore, a need currently exists to develop and deploy novel strategies aimed at reducing or preventing the emergence of CWD in farmed NA elk.

## Materials and methods

### Study overview

In the present study, we investigate differential susceptibility to CWD among farmed NA elk by performing next-generation sequencing with variant prediction for the construction and validation of a medium-density custom variant array using genomic DNA samples from farmed elk. Thereafter, we use the custom array, which includes *PRNP* genotypes, to conduct genome-wide association analyses (GWAAs) with marker-based heritability estimates. Finally, to conclude the present study, we utilize the genome-wide variant data to deploy genomic prediction equations with *k*-fold cross-validation to assess the potential for developing a genomically estimated CWD mitigation program.

### Animal resources, CWD diagnostics, and DNA isolation

Hair samples from farmed NA elk (*n* > 400) were available within an existing repository for DNA isolation and genome sequencing for variant prediction. Tissue samples (*n* = 1,055) from farmed NA elk (both sexes) were collectively available within an existing USDA APHIS repository created via federal CWD surveillance activities (*n* = 18), including depopulations of CWD-positive herds (USDA APHIS, Fort Collins, CO), a similar Canadian Food Inspection Agency repository (*n* = 858), and a research repository (*n* = 179) at Midwestern University (Glendale, AZ, USA). The available samples included both CWD-positive (*n* = 394) and CWD nondetect (*n* = 661) elk (Haley and Richt 2017 ), with geographic representation that included farms located in the United States and Canada. All US diagnostic classifications were based upon confirmatory immunohistochemistry (i.e. IHC of lymph node, obex, and/or rectal biopsy), as implemented and performed at the USDA National Veterinary Services Laboratories (NVSL) in Ames, Iowa (Thomsen et al. 2012; Haley and Richt 2017). All Canadian diagnostic classifications (i.e. all postmortem CWD positives) were confirmed by IHC at the Canadian Food Inspection Agency National and World Organization for Animal Health Reference Laboratory for CWD (Ottawa, Ontario, CAN). Genomic DNA for next-generation sequencing and variant prediction was isolated from hair follicles obtained from farmed NA elk (*n* = 400) using the MasterPure Complete DNA & RNA Purification Kit (MC85200), as recommended by the manufacturer (Lucigen; Middleton, WI, USA). Thereafter, the resulting DNA samples were quantified and evaluated for purity (260/280 ratio) via Nanodrop (Thermo Fisher). Genomic DNA from farmed elk tissue samples (*n* = 1,055) was isolated using the LGC sbeadex tissue purification kit (LGC) with automation at GeneSeek Neogen (Lincoln, NE).

### Paired-end libraries and sequencing

DNA pooling is known to be effective for variant prediction, but also for directly enabling downstream genotyping strategies (Seabury et al. 2011, 2020, 2022). Therefore, a single DNA pool was created for farmed NA elk, for the purpose of pair-end library construction and sequencing. Specifically, farmed elk DNAs (*n* = 400; from hair follicles) with concentrations ≥ 15 ng/µL were used to construct a sequencing pool by targeting 50 ng of genomic DNA per individual, as previously described (Seabury et al. 2020), with the pooled elk DNA further purified using next-generation sequencing solid-phase reversible immobilization (SPRI) magnetic bead technology (Omega Bio-tek Inc, Norcross, GA). Thereafter, 8 independent paired-end (PE) libraries were individually prepared using 25 ng of the purified elk genomic DNA, as implemented in a custom, automated, and miniaturized version of the PerkinElmer (Waltham, MA) NEXTFLEX Rapid XP kit (Johnson et al. 2019). Briefly, purified elk genomic DNA was enzymatically fragmented for 6 min (PerkinElmer, Waltham, MA), ligated to unique dual-indexed barcodes, SPRI size selected (520 to 720 bp), and amplified with 10 PCR cycles; using one-tenth of manufacturer-recommended volumes for the enzymatic steps. The 8 PE libraries were then diluted with elution buffer (Omega Bio-tek Inc, Norcross, GA) to a final concentration of 2 ng/µL and pooled by equivolume. The PE sequencing pool was then loaded and sequenced on 1 lane of an Illumina NovaSeq S4 v1.5 XP using the 2 × 150-bp method.

### Sequence analysis and Affymetrix Axiom array design

Illumina sequences from farmed NA elk were trimmed for quality and adapters using CLC Genomics Workbench 21.0.4 (Qiagen), as previously described (Halley et al. 2014; Halley et al. 2015; Sollars et al. 2017; Seabury et al. 2020). Thereafter, trimmed reads produced from 6 of the 8 PE libraries were mapped to the elk (*Cervus canadensis*) reference genome assembly (GCA_019320065.1; ASM1932006v1; https://www.ncbi.nlm.nih.gov/datasets/genome/GCF_019320065.1/) using the CLC Genomics Workbench 21.0.4 reference mapping algorithm (Seabury et al. 2011, 2020; Halley et al. 2014; Halley et al. 2015; Sollars et al. 2017). Reference mapping was limited to 6 PE libraries (>2.298 billion PE reads) for computational expediency and stability given 0.5 TB of RAM allocated to the mapping algorithm. Thereafter, genome-wide variant prediction was performed using a probabilistic approach implemented within CLC Genomics Workbench 21.0.4 (Halley et al. 2014; Halley et al. 2015; Oldeschulte et al. 2017; Sollars et al. 2017; Seabury et al. 2020). Notably, the variant prediction algorithm uses quality scores to estimate error probabilities, with these probabilities used to determine the most likely allele combination per nucleotide site, thus allowing for a user-specified minimum probability threshold (*P* ≥ 0.95) for variant prediction, and variant quality scores (Halley et al. 2014; Halley et al. 2015; Oldeschulte et al. 2017; Sollars et al. 2017; Seabury et al. 2020). Additional filters and variant prediction parameters were similar to those previously described (Seabury et al. 2011 , 2020; Oldeschulte et al. 2017), with the probabilistic approach producing evidence for 6,745,261 variants (*P* ≥ 0.95; minor allele frequency ≥ 0.01). Elk genome-wide variants were exported from CLC as a single variant call format file (VCF) and used for downstream array design, which included filtering according to the Affymetrix Axiom myDesign technical guidelines (https://www.thermofisher.com/order/catalog/product/000799?SID=srch-srp-000799). All elk genome-wide variants were evaluated by Affymetrix scoring (i.e. by pconvert, best_pconvert; recommended, neutral, or not recommended), to enable the design of a final Affymetrix Axiom 200K variant array. Initially, 200,000 elk genome-wide variants that scored favorably (and known *PRNP* variants) were selected for array fabrication. The final custom elk array comprised 184,303 elk genome-wide variants that were favorably scored (*n* = 184,300 recommended; *n* = 3 neutral) and 21 that were not recommended, with all 184,324 used for final fabrication, including known variants within the *PRNP* gene (i.e. codon 132Met→Leu, promoter) (O’Rourke et al. 1999; Seabury et al. 2007; White et al. 2010).

### Affymetrix Axiom array genotyping

Affymetrix Axiom 200Kv2 elk genotyping was performed at GeneSeek Neogen using the established Affymetrix best practices workflow, with genotypes delivered in text format. The relevant Affymetrix quality control thresholds were DQC ≥ 0.82, QC call rate ≥ 95%, and average call rate for passing samples used in all analyses > 98%. Collectively, 910 NA farmed elk samples with additional metadata (i.e. sex, age, geographic region of origin, and *PRNP* genotypes) passed all Affymetrix QC filters, each with 184,324 variant array genotypes for analysis, including *PRNP* genotypes (i.e. codon 132, promoter) and Y chromosome markers that were used to verify the recorded sex. Elk with existing *PRNP* codon 132 genotypes (*n* = 886; i.e. by Sanger sequencing or by TaqMan probe) were reconciled with the array genotypes for additional quality control.

### Quality control, GWAA, and genomic prediction with cross-validation

Following sample call rate filtering (<95% excluded), pairwise identity by state (IBS) distances were computed to identify duplicate NA farmed elk samples (Seabury et al. 2020). Six samples were duplicated and identified by IBS estimates (with *n* = 5 treated as process controls). For all duplicates, only the sample with the highest call rate was retained for further analyses, as previously described (Seabury et al. 2020 ). Additional quality control analyses and filtering were as follows: variant filtering by call rate (>15% missing excluded), minor allele frequency (MAF < 0.01 excluded), polymorphism (monomorphic SNPs excluded), and Hardy–Weinberg equilibrium (HWE; excludes SNPs with HWE *P* < 1e-25 for autosomes and *P* < 1e-50 for the X chromosome), thus yielding 173,674 genome-wide variants for all final analyses. All GWAAs and genomic predictions with *k*-fold cross-validation were performed on 904 NA farmed elk with metadata including sex, age (i.e. mean for missing), geographic region of origin (i.e. United States and Canada), and CWD diagnostics. CWD phenotypes used in all analyses were as follows: CWD binary (0 = nondetect, 1 = lymph node-positive and/or obex positive). All NA farmed elk GWAAs were performed using a mixed linear model with genomic relationship matrix (GRM) (VanRaden 2008) and variance component estimates, as described and implemented in Efficient Mixed-Model Association eXpedited (EMMAX), and executed in SVS v8.9.0 (Golden Helix), with all genotypes coded as 0, 1, or 2, based on the incidence of the minor allele (Kang et al. 2010 ; Segura et al. 2012; Seabury et al. 2017, 2020, 2022, 2023; Smith et al. 2019). The NA farmed elk mixed model can be generally specified as y=Xβ+Zu+∈, where *y* represents a *n* × 1 vector of observed elk CWD phenotypes, *X* is a *n* × *f* matrix of fixed effects, *β* is a *f* × 1 vector representing the coefficients of the fixed effects, *u* represents the unknown random effect, *Z* is a *n* × *t* matrix relating the random effect to the elk CWD phenotypes of interest, and ∈ represents the residual errors (Kang et al. 2010; Segura et al. 2012; Seabury et al. 2017, 2020, 2022, 2023; Smith et al. 2019). Herein, we must assume that $Var(u)=\sigma_{g}^{2}K$ and $Var(\in )=\sigma_{e}^{2}I$, such that $Var(y)=\sigma_{g}^{2}ZKZ^{\prime }+\sigma_{e}^{2}I$, but in this study, *Z* represents the identity matrix *I*, and *K* represents a relationship matrix of all NA farmed elk with acceptable call rates in the present study. Moreover, to solve the mixed model equation, we must first estimate the variance components (i.e. $\sigma_{g}^{2}$ and $\sigma_{e}^{2}$) as previously described (Kang et al. 2010 ; Segura et al. 2012; Seabury et al. 2017, 2020, 2022, 2023; Smith et al. 2019). The relevant variance components were estimated using the restricted maximum likelihood-based Efficient Mixed-Model Association (EMMA) approach (Kang et al. 2010), with stratification accounted for and controlled using a GRM (*G*) (VanRaden 2008); which was computed from the NA farmed elk genotypes. GRM heritability estimates ($\left[h^{2}=\sigma_{g}^{2}/(\sigma_{g}^{2}+\sigma_{e}^{2})\right]$) for differential susceptibility to CWD in NA farmed elk were produced as previously described (Kang et al. 2010 ; Segura et al. 2012 ; Seabury et al. 2017, 2020, 2022, 2023; Smith et al. 2019). Additionally, because the probability of CWD infection may increase with age (Grear et al. 2006 ; Allen et al. 2025), and given the potential for CWD to disparately affect male and female NA farmed elk in different geographic regions (Grear et al. 2006; Edmunds et al. 2016; Miller et al. 2020; Conner et al. 2021), the following fixed-effect covariates were specified for comparison of two GWAAs: sex, age, and geographic region of origin (i.e. United States and Canada) vs no fixed-effect covariates. Genomic inflation factors were estimated in SVS v8.9.0 (Golden Helix) as follows: pseudo-lambda = log10(median observed *P*-value)/log10(median expected *P*-value).

To assess the potential for incorporating genome-wide variant information into an organized breeding program that reduces the risk of CWD infection among NA farmed elk, we performed genomic prediction with *k*-fold cross-validation (*k* = 3; *k* = 5). For all such analyses, we used genomic best linear unbiased prediction (GBLUP) as previously described (Taylor 2014) and executed in SVS v8.9.0 (Golden Helix), with the variance components again estimated using the restricted maximum likelihood-based EMMA approach (Kang et al. 2010) in conjunction with a GRM (*G*) (VanRaden 2008; Taylor 2014; Seabury et al. 2020 ; Seabury et al. 2023). For all elk GBLUP analyses, the general mixed model equation can be specified as $y=X_{f}B_{f}+u+\in$, across *n* elk samples where fixed effects specified as *B_f_* include the intercept and any additional covariates (i.e. sex, age, and geographic region of origin such as United States or Canada), but where $Var(\in )=\sigma_{e}^{2}I$, as described above, and where the random effects *u* are the additive genetic merits (i.e. the genomically estimated breeding values or GEBVs) for these elk samples, which are produced from *m* elk genetic markers as *u* = *Mα*, where *M* is a *n* × *m* matrix, and *α* is a vector, where *α_k_* is the allele substitution effect (ASE) for elk marker *k* (Seabury et al. 2020). For the present study, we used overall normalization for matrix *M*, as implemented in SVS v8.9.0 (Golden Helix), and investigated solutions with sex corrections (i.e. full dosage compensation) as previously described (Taylor 2014 ; Seabury et al. 2020), to produce GEBVs for all NA farmed elk samples as well as estimates of ASE for all filtered array markers. Given a general workflow where all training set samples will precede any validation set, we define Z=[I|0], where the width and height of *I* are represented as *n_t_*, the width of the zero matrix is represented as *n_v_*, and the height of the zero matrix is *n_t_*, as previously described (Seabury et al. 2020, 2023). We can then further define *u*, *X_f_*, and *y* according to their origins (i.e. the elk training set vs the validation set) as $u=\left[\begin{array}{c} u_{t}\\ u_{v} \end{array}\right]$, $X_{f}=\left[\begin{array}{c} X_{\;ft}\\ X_{\;fv} \end{array}\right]$, and $y=\left[\begin{array}{c} y_{t}\\ y_{v} \end{array}\right]$, and compute a GRM (*G*) (VanRaden 2008) using all NA farmed elk samples for use with the EMMA technique (Kang et al. 2010), to deploy a mixed model for the elk training set as follows: $y_{t}=X_{ft}B_{f}+Zu+\in_{t}$, where $Var(u)=\sigma_{G}^{2}G$ and $Var(Zu)=\sigma_{G}^{2}ZGZ^{\prime }$ (Seabury et al. 2020, 2023). Thereafter, we predict the elk validation set CWD phenotypes as ${\hat{y}}_{v}=X_{fv}{\hat{B}}_{f}+{\hat{u}}_{v}$, from the intercept and any validation covariates *X_fv_* as well as the predicted values of ${\hat{u}}_{v}$, as previously described in farmed white-tailed deer (Seabury et al. 2020). Additional formulae, methods, and further documentation are available online as follows: https://doc.goldenhelix.com/SVS/latest/svsmanual/mixedModelMethods/overview.html#gblupproblemstmt; https://www.goldenhelix.com/docs/SVS/latest/svsmanual/mixedModelMethods/gblup_analysis.html?highlight=genomic%20prediction. To our best knowledge, GEBVs have not been estimated or utilized in farmed elk prior to the present study, and as such, the concept of individual additive genetic merits within the context of CWD may not be highly intuitive or easily understood by NA elk farmers or relevant regulatory agencies. However, the predicted elk CWD binary phenotypes can be easily interpreted as estimates of differential susceptibility to CWD. More specifically, using the GBLUP approach described above, we predict the CWD binary phenotypes across a range of values (i.e. 0 to 1) using SVS v8.9.0 (Golden Helix), with predicted values ≥ 0.5 considered as “1,” and < 0.5 as “0,” thereby enabling the computation of relevant summary statistics for binary classifications, as previously described (Seabury et al. 2020). Consistent with a prior study involving farmed white-tailed deer (Seabury et al. 2020), analytical support for rounding in the present study is similarly apparent by the histograms representing the frequency distributions of the predicted elk CWD binary phenotypes (Supplementary Fig. 1), the relevant elk GEBVs (Supplementary Fig. 2), and the relationship between them (Supplementary Fig. 3); with a discernible break that occurs at 0.50 (Supplementary Figs. 1 to 3). Relevant binary summary statistics for genomic predictions using GBLUP in conjunction with *k*-fold cross-validation (*k* = 3; *k* = 5; *n* = 50 iterations) were computed in SVS v8.9.0 (Golden Helix) as follows: area under the curve as $AUC=\frac{U_{1}}{n_{1}n_{2}}$, where *n*_1_ is the number of observations (i.e. the sample size) with elk CWD binary phenotypes of 0, *n*_2_ is the number of observations (i.e. the sample size) with elk CWD binary phenotypes of 1, and $U_{1}=R_{1}-\frac{n_{1}(n_{1}+1)}{2}$, where *R*_1_ is the sum of the ranks for the predicted binary elk CWD phenotypes with actual phenotypes of 0 (from elk CWD diagnostics); Mathews correlation coefficient was computed as $MCC=\frac{TP\cdot TN-FP\cdot FN}{\sqrt{\left(TP+FN)\cdot (FP+TN)\cdot (TP+FP)\cdot (FN+TN\right)}}$, where *TP* represents the number of true CWD positives (from elk CWD diagnostics), *TN* represents the number of true CWD nondetects (from elk CWD diagnostics), *FP* represents the number of false positives, and *FN* represents the number of false nondetects among the predicted elk CWD binary phenotypes; genomic prediction accuracy was estimated as $ACC=\frac{TP+TN}{TP+FN+FP+TN}$; sensitivity (true-positive rate) was estimated as $TPR=\frac{TP}{TP+FN}$; specificity (true-negative rate) was estimated as $SPC=\frac{TN}{FP+TN}$; root mean square error was computed as $RMSE=\sqrt{\frac{\sum_{i=1}^{n}{\left(y_{i}-{\hat{y}}_{i}\right)}^{2}}{n}}$, as previously described (Seabury et al. 2020, 2023).

### Randomizing and blinding

All GBLUP-based *k*-fold (*k* = 3; *k* = 5) cross-validations (i.e. CWD binary phenotype) were performed using automated random sampling to define the elk validation set (i.e. to predict on) and the elk training set, for each of the specified values of *k* across 50 iterations (Seabury et al. 2020, 2023; Seabury 2023 ).

## Results and discussion

**Fig. 1.**
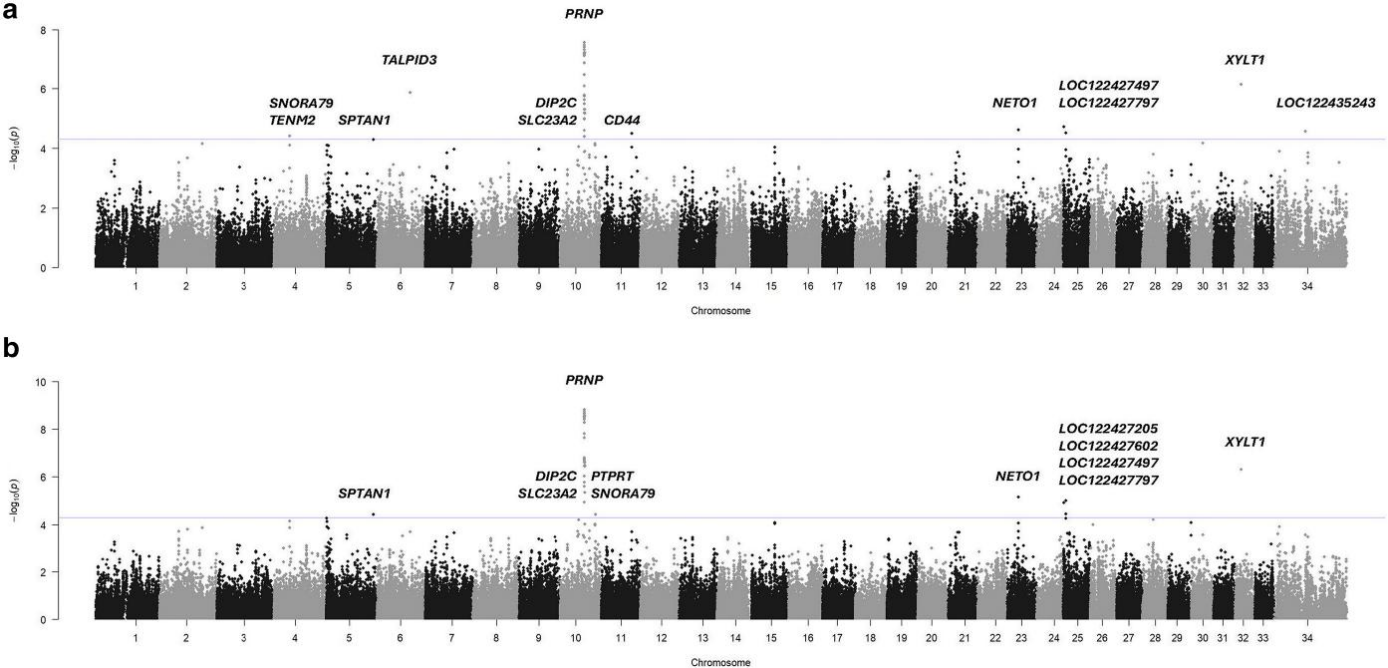
EMMAX binary case–control (0, 1) GWAAs for CWD in farmed NA elk (*C. canadensis*). The Manhattan plots depict −log10 *P*-values for variable elk markers on the *y* axis, and their genomic positions, respectively, on the *x* axis, where the X chromosome = 34 (ASM1932006v1). All analyses include diagnostically confirmed CWD-positive (*n* = 357) and CWD nondetect (*n* = 547) elk, and marker-based GRM heritability estimates ($\left[h^{2}=\sigma_{g}^{2}/(\sigma_{g}^{2}+\sigma_{e}^{2})\right]$) (Kang et al. 2010; Segura et al. 2012; Seabury et al. 2017, 2020, 2022, 2023; Smith et al. 2019). a) EMMAX GWAA for CWD with no fixed-effect covariates, high GRM heritability estimates ($h^{2}=0.678\pm 0.056$), and relevant positional candidate genes, whereby the estimated genomic inflation factor (pseudo-lambda) = 0.9945. b) EMMAX GWAA for CWD with fixed-effect covariates (sex, age, and geographic region of origin), high GRM heritability estimates ($h^{2}=0.630\pm 0.061$), and relevant positional candidate genes, whereby the estimated genomic inflation factor (pseudo-lambda) = 0.9905.

Bioinformatic analyses focusing on Illumina PE sequencing data from 6 pooled DNA libraries, including reference mapping and variant prediction, provided the necessary foundation for the construction of a custom Affymetrix Axiom 200K variant array for farmed NA elk (*C. canadensis*) (see Materials and methods). Thereafter, we produced a final genomic data set consisting of Affymetrix Axiom 200K array genotypes (*n* = 173,674 variable markers) for 904 farmed NA elk (United States and Canada) that were diagnostically classified as CWD positive (*n* = 357) and CWD nondetect (*n* = 547) (see Materials and methods). GWAAs were conducted using a mixed linear model (*n* = 173,674 variable markers; *n* = 904 farmed NA elk), both with and without additional fixed-effect covariates (i.e. sex, age, and geographic region of origin), and included a GRM with variance component analysis, thereby producing marker-based heritability estimates for differential susceptibility to CWD, as implemented in EMMAX (Kang et al. 2010; Segura et al. 2012; Seabury et al. 2020, 2022). Similar to previous reports for farmed white-tailed deer (Seabury et al. 2020, 2022), GRM heritability estimates produced for differential susceptibility to CWD in farmed NA elk were high (i.e. $h^{2}=0.630\pm 0.061-0.678\pm 0.056$) across both GWAA models evaluated in the present study (Fig. 1), with natural differences in susceptibility primarily modulated via genetic control (i.e. as a polygenic trait with environmental influence), as opposed to the effects of a single codon (i.e. *PRNP* codon 132) or biological luck with respect to exposure (Allen et al. 2025). Moreover, while we unequivocally confirm *PRNP* as one major risk locus for CWD in farmed NA elk (i.e. the promoter; codon 132Met→Leu; intron 1, exon 2), we also detected 17 additional variants outside of *PRNP* (Fig. 1; Supplementary Table 1) that met a nominal significance threshold (*P* ≤ 5E-05) for polygenic traits (Wellcome Trust Case Control Consortium 2007; Seabury et al. 2017; Seabury et al. 2020; Seabury et al. 2022). Across both GWAA models, the largest-effect markers [i.e. proportion of phenotypic variance explained {PVE}] were detected within the *PRNP* promoter (PVE ≤ 0.398; *P* ≤ 2.743E-08) and for *PRNP* codon 132 (PVE = 0.032; *P* ≤ 6.090E-08; missense Met→Leu; Fig. 1; Supplementary Table 1), which is compatible with previous studies on elk *PRNP* variation and CWD risk (O’Rourke et al. 1999; Seabury et al. 2007). Altogether, 17 elk possessed the *PRNP* codon 132Leu/Leu genotype, and 6 of those elk were diagnostically confirmed as being CWD positive, thereby underscoring the need to account for the additive polygenic effects outside of *PRNP* when contemplating future breeding programs. We also detected strong nonrandom associations [i.e. high linkage disequilibrium {LD}] between many farmed NA elk *PRNP* promoter variants associated with CWD, and natural variation within *PRNP* codon 132 (Supplementary Table 1). However, LD was not complete between all *PRNP* promoter variants and *PRNP* codon 132 (Supplementary Table 1), as was initially observed in a prior study encompassing less genetic diversity and smaller sample sizes (Seabury et al. 2007). Collectively, our *PRNP* results highlight the molecular potential for the combined effects of qualitative (i.e. structural variation in the cellular prion protein) and quantitative mechanisms (i.e. variation in *PRNP* expression) as important risk factors influencing differential susceptibility to CWD in farmed NA elk (Scott et al. 1989; Westaway et al. 1991; Büeler et al. 1993; Manson et al. 1994; O’Rourke et al. 1999; McCormack et al. 2002; Haigh and Brown 2006; Bratosiewicz-Wasik et al. 2007; Seabury et al. 2007; Bratosiewicz-Wąsik et al. 2012). Notably, while the control of cellular prion protein expression (i.e. *PRNP*→PrP^C^) is considered somewhat complex across tissues, it is known to be influenced by the *PRNP* promoter and/or intron 1, with the rate of disease progression in a susceptible host often directly related to the quantitative expression levels of endogenous PrP^C^ (Scott et al. 1989; Westaway et al. 1991; Büeler et al. 1993; Manson et al. 1994; Vilotte et al. 2001; McCormack et al. 2002; Castilla et al. 2004; Safar et al. 2005; Sander et al. 2005; Scott et al. 2005; Haigh and Brown 2006; Bratosiewicz-Wasik et al. 2007; Seabury et al. 2007). For these reasons, future molecular studies should jointly evaluate the functional implications of elk *PRNP* promoter variation together with missense variation at *PRNP* codon 132, in CWD-appropriate elk tissues.

**Fig. 2.**
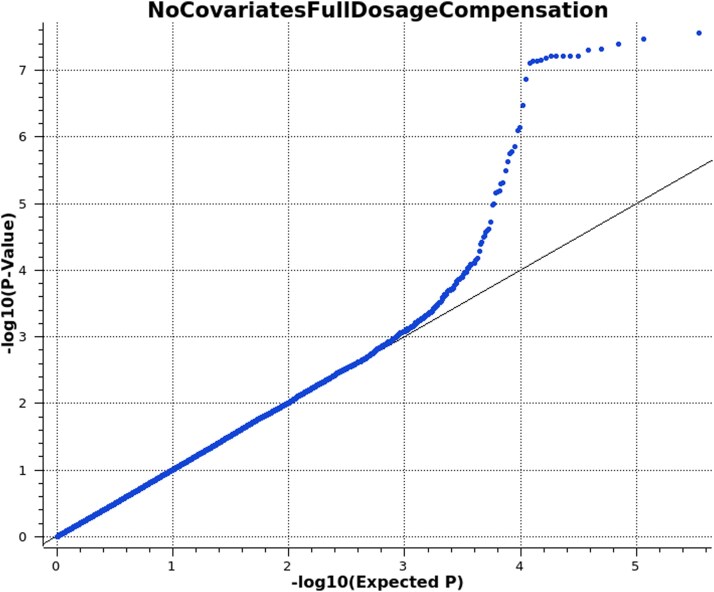
*P*-*P* plot for an EMMAX binary CWD GWAA, which included full dosage compensation, but without additional fixed-effect covariates. The *x* axis represents the expected −log10 *P*-values, and the *y* axis represents the observed −log10 *P*-values produced via EMMAX.

**Fig. 3.**
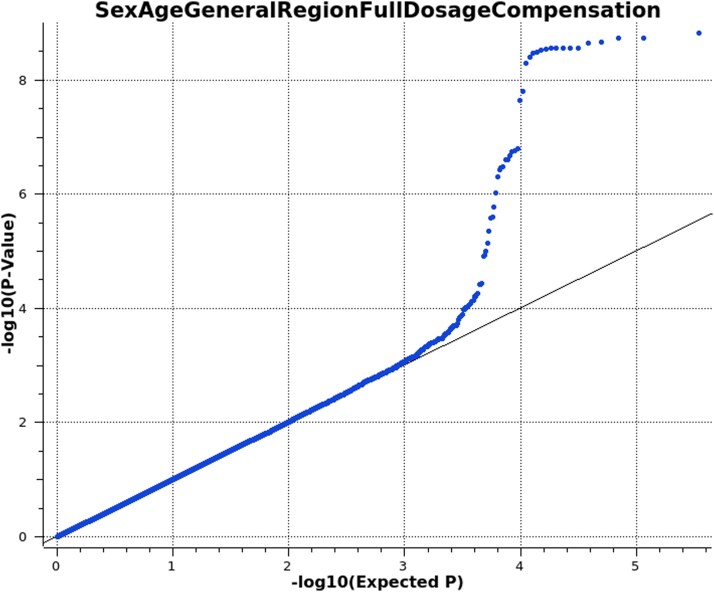
*P*-*P* plot for an EMMAX binary CWD GWAA, which included full dosage compensation and relevant fixed-effect covariates (i.e. sex, age, and geographic region of origin). The *x* axis represents the expected −log10 *P*-values, and the *y* axis represents the observed −log10 *P*-values produced via EMMAX.

Beyond *PRNP*, and owing to the polygenic nature of differential susceptibility to CWD in farmed NA elk (Fig. 1; Supplementary Table 1; $h^{2}=0.630\pm 0.061-0.678\pm 0.056$), significant genome-wide variants (*P* ≤ 5E-05) were also detected on 8 additional elk chromosomes (i.e. 4, 5, 6, 11, 23, 25, 32, and X), and outside of *PRNP* on elk chromosome 10 (i.e. prioritizing *PTPRT*), with *PTPRT* being important for the regulation of synaptic formation and neuronal development (Lee 2015). These results are compatible with prior analyses performed in mice and farmed NA white-tailed deer, where incubation times, disease progression, or general risk for transmissible spongiform encephalopathies were largely influenced by a genetic architecture independent of *PRNP* (Iyegbe et al. 2010; Seabury et al. 2020, 2022). Likewise, when at least 1 functional *PRNP* allele exists within the mammalian genome, the polygenic nature of prion diseases has also been noted in humans, sheep, and cattle (Moreno et al. 2008; Mead et al. 2009; Murdoch et al. 2010, 2011; Jones et al. 2020). Positional candidate genes identified on elk chromosomes 4 (*TENM2*) and 5 (*SPTAN1*) have been associated with various aspects of neural development including axon guidance and synaptogenesis, respectively, with mutations in *SPTAN1* previously associated with epileptic encephalopathy as well as other neurological issues including ataxia (Silva et al. 2011; Morsy et al. 2023). However, recent studies have also identified associations between *TENM2* and enhanced risk for Alzheimer's disease (Sáez et al. 2019; Xu et al. 2022). For elk variants on chromosomes 6 (*TALPID3*) and 11 (*CD44*), it is interesting to note that mutations in *TALPID3* have been associated with Joubert syndrome (i.e. including possible hypotonia and ataxia), which primarily affects the cerebellum and brainstem, whereas *CD44* is well-known to be upregulated with the early onset of central nervous system prion disease pathology (Tortosa et al. 2011 ; Bashford and Subramanian 2019; Bradford et al. 2019; Fraser and Davey 2019; Bradford et al. 2024). The marker-based association noted on elk chromosome 23 (Supplementary Table 1; Fig. 1) is proximal to *NETO1*, which is known to be involved in normal neural function including synaptic plasticity, whereas synaptic dysfunction is an established hallmark of neurodegenerative prion diseases (Senatore et al. 2013; Li et al. 2019). While multiple associations were detected on elk chromosome 25, thereby prioritizing several long noncoding RNA genes of unknown function (Supplementary Table 1; Fig. 1), the most intriguing association prioritized 2 olfactory receptor genes as positional candidates for differential susceptibility to CWD. Interestingly, while the gastrointestinal tract is often considered the primary point of entry for many prion infections, the olfactory mucosa is also considered an important point of entry (Nichols et al. 2013), with progressive olfactory dysfunction being common among many neurodegenerative diseases (Crowell et al. 2015; Rey et al. 2018). Relevant to CWD in farmed NA elk, prion infection of olfactory receptor neurons is known to facilitate the shedding of infectious material into the nasal fluids, which is pertinent to CWD transmission (Bessen et al. 2010; Haley et al. 2016). Finally, the association detected on elk chromosome 32 (*XYLT1*) prioritizes a gene that is known to be involved in synthesizing proteoglycans and more specifically, the addition of glycosaminoglycan (GAG) side chains to proteoglycans, whereas the association signal on the elk X chromosome was found to prioritize a long noncoding RNA (*LOC122435243*) of unknown function as well as *NUDT11* (Supplementary Table 1; Fig. 1). While *NUDT11* has no known function in prion disease pathology, a recent study produced overt statistical evidence suggesting that variability in the efficiency of GAG catabolism may in fact influence differential susceptibility to CWD in farmed NA white-tailed deer, with the presence of sulfated GAGs first observed to colocalize with amyloid plaques in CWD-affected mule deer more than 30 years ago (Guiroy et al. 1991; Seabury et al. 2022). For these reasons, *XYLT1* should be further investigated in relation to the pathophysiology of CWD in farmed NA elk. Detailed summary data for all GWAAs and positional candidate genes (Supplementary Table 1; Fig. 1) are provided in the supplementary information (Additional Files 1 and 2 in DRYAD doi: https://doi.org/10.5061/dryad.p2ngf1w57); with corresponding *P*-*P* plots demonstrating effective mitigation of population stratification depicted in Figs. 2 and 3. Likewise, genomic inflation factors computed for both GWAAs (0.9945; 0.9905; Fig. 1) provided no evidence for spurious associations resulting from population substructure.

To investigate the potential impact of developing a genomically estimated breeding program to reduce the incidence of CWD in farmed NA elk, we used GBLUP together with *k*-fold cross-validation and random sampling (Taylor 2014; Seabury et al. 2020; Seabury et al. 2023). Specifically, farmed NA elk data (i.e. array genotypes, CWD diagnostic phenotypes, and other metadata) were randomly segmented and assigned to *k*-subsamples (i.e. *k* = 3; *k* = 5), and one of the subsamples was then designated to serve as the validation set for genomic prediction (i.e. within a discrete fold); with the GBLUP model fit using the remaining (i.e. *k* −1) subsamples within the same fold (i.e. as the training data), until all subsamples were used for both training and prediction (Seabury et al. 2020, 2023; Seabury 2023). To produce relevant summary statistics for the farmed NA elk genomic predictions, all cross-validations with random sampling were run for 50 iterations, whereby each iteration consisted of either three (*k* = 3) or five (*k* = 5) folds (see Table 1; Materials and methods; Additional Files 3 to 6 in DRYAD). Binary GBLUP models fit with no fixed-effect covariates (*k* = 3, *k* = 5) produced high mean estimates for AUC (≥0.8352), genomic prediction accuracies (≥0.7889), and specificities (≥0.8595), with small standard deviations, but lower mean sensitivities (≤0.6807). All summary statistics were very similar to those previously produced for farmed NA white-tailed deer (Seabury et al. 2020 ), with both studies suggesting that false negatives may potentially pose the greatest challenge for reducing the incidence or prevalence of CWD via genomic prediction (Table 1; see Materials and methods). However, it should also be noted that mean sensitivities were slightly higher in farmed NA elk, as compared to farmed NA white-tailed deer (Seabury et al. 2020). When binary GBLUP models were fit using sex, age, and geographic region of origin as fixed-effect covariates, similar summary statistics were obtained (*k* = 3; *k* = 5; Table 1). Across all genomic predictions with *k*-fold cross-validation, some false-positive predictions were also observed (*k* = 3; *k* = 5; Table 1), which were most likely due to underlying genomic susceptibility, early stages of disease (i.e. CWD nondetect diagnostically), and/or differences in exposure (Tsairidou et al. 2014; Seabury et al. 2020). Relevant to the goals of the present study, genomic predictions deployed herein were never intended to represent a diagnostic assay, but rather, they represent a robust foundation for implementing a genetic improvement program among farmed NA elk with respect to differential susceptibility to CWD.

**Table 1.** Summary of CWD genomic predictions in farmed NA elk (C. canadensis).

*k*-fold subsample	GBLUP model covariates	Mean AUC (*SD*)^a^	Mean Matthews coefficient (*SD*)	Mean genomic prediction accuracy (*SD*)	Mean sensitivity (*SD*)	Mean specificity (*SD*)	Mean RMSE (*SD*)^b^
*k* = 3	None	0.8352 (0.0078)	0.5524 (0.0174)	0.7889 (0.0082)	0.6807 (0.0152)	0.8595 (0.0122)	0.4008 (0.0043)
*k* = 3	Sex, age, geographic region	0.8371 (0.0074)	0.5548 (0.0179)	0.7908 (0.0082)	0.6473 (0.0156)	0.8845 (0.0101)	0.3983 (0.0040)
*k* = 5	None	0.8408 (0.0061)	0.5703 (0.0144)	0.7971 (0.0067)	0.6965 (0.0117)	0.8628 (0.0077)	0.3968 (0.0036)
*k* = 5	Sex, age, geographic region	0.8431 (0.0055)	0.5781 (0.0147)	0.8014 (0.0068)	0.6700 (0.0109)	0.8872 (0.0079)	0.3947 (0.0034)

^a^Area under the curve (AUC; Wilcoxon–Mann–Whitney method; see Materials and methods).

^b^Root mean square error (RMSE; see Materials and methods).

### Conclusion

While prior elk CWD studies have exclusively focused on genetic variation within the *PRNP* gene, herein we clearly demonstrate that differential susceptibility to CWD in farmed NA elk is a polygenic trait with environmental variance ($h^{2}=0.630\pm 0.061-0.678\pm 0.056$). Likewise, we also demonstrate that natural genetic variation (i.e. on a genome-wide basis) can be used to produce accurate genomic predictions for CWD risk (AUC = 0.8352 ─ 0.8431; ACC = 0.7889 ─ 0.8014), thereby providing a molecular foundation for genetic improvement of farmed NA elk with respect to reducing the incidence of CWD. We also note many similarities between the genomic architecture of CWD risk in farmed NA elk and farmed NA white-tailed deer, where *PRNP* genotyping alone cannot be expected to facilitate an eradication program in either species. Moreover, it should also be noted that all *PRNP* codon 132 Leu/Leu elk are not created equal with respect to the additive genetic merits of differential susceptibility to CWD; which is conceptually well aligned with the results of similar studies for farmed NA white-tailed deer.

## Supplementary Material

jkag082_Supplementary_Data

## Data Availability

Accession codes are as follows: Illumina sequence data (SRA: PRJNA1426504) and genotype and phenotype data (DRYAD doi: https://doi.org/10.5061/dryad.p2ngf1w57). Supplemental material available at *G3* online.
