# Supplementary material for: Accurate genomic predictions for chronic wasting disease in North American elk
Source: G3 (Bethesda). 2026 Mar 31;16(8):jkag082. doi: 10.1093/g3journal/jkag082 (PMC13439932; doi:10.1093/g3journal/jkag082)
Supplement: Supplementary file 1 [file jkag082_supplementary_data.zip › Supplemental_Figures_1-3_G3-2025-406493.pdf]

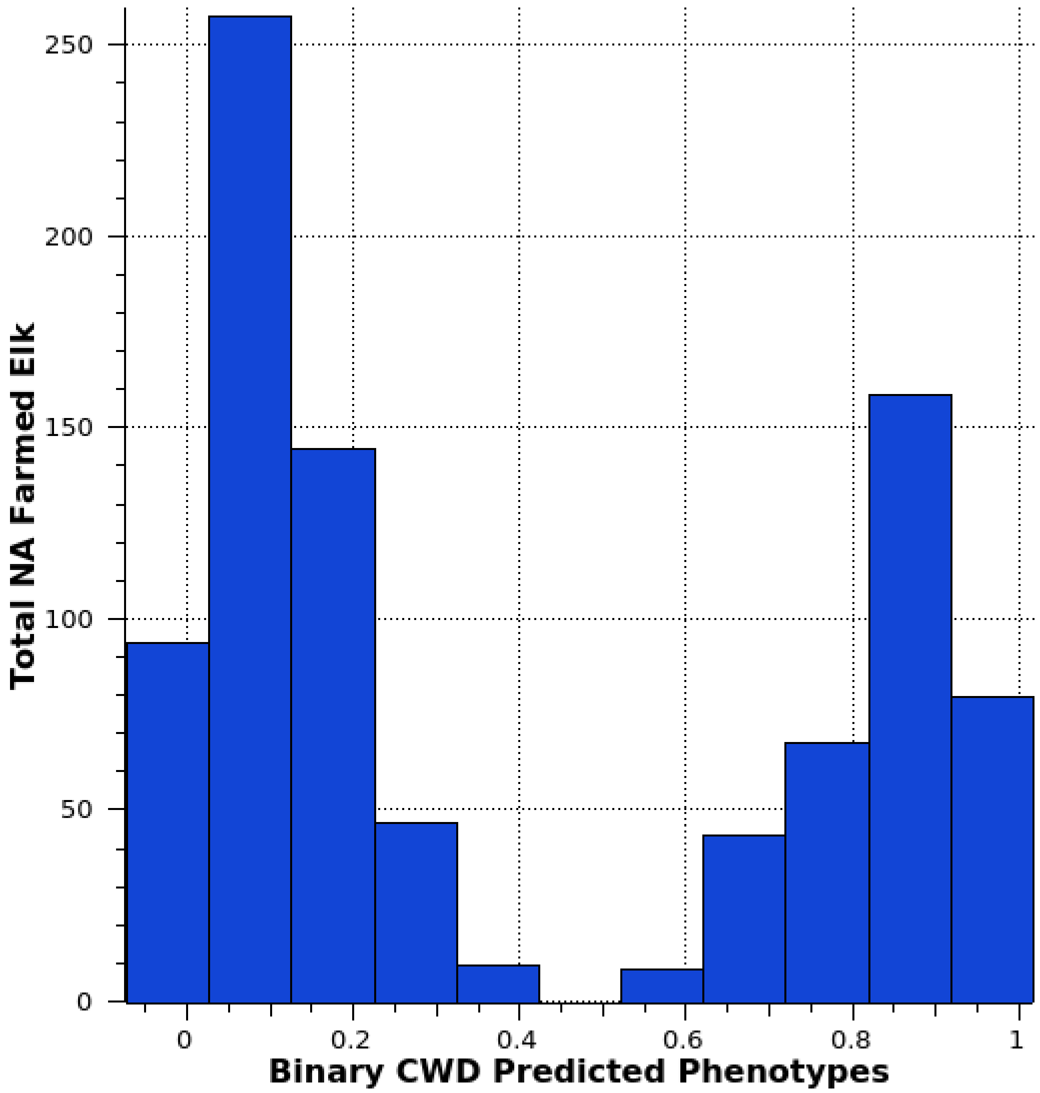

Figure S1 (A). Histogram of binary CWD phenotypes predicted using GBLUP with full dosage compensation and no fixed-effect covariates (n = 173,674 variants) for 904 farmed North American (NA) elk (*Cervus canadensis*).

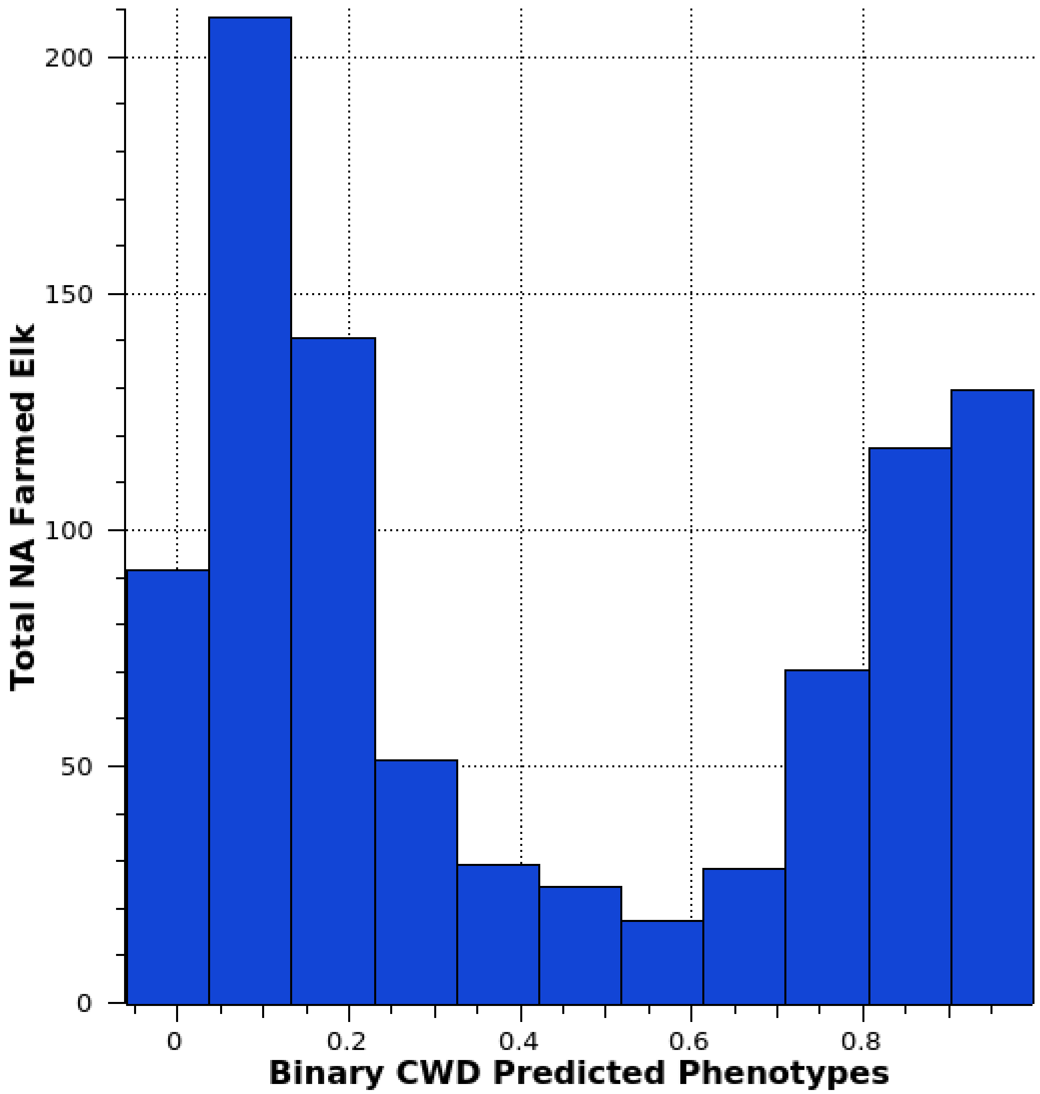

Figure S1 (B). Histogram of binary CWD phenotypes predicted using GBLUP with full dosage compensation and no fixed-effect covariates (n = 173,674 variants) for 904 farmed North American (NA) elk (*Cervus canadensis*). For this analysis we randomly masked (i.e., set as missing) the known binary CWD diagnostic phenotypes for 181 farmed NA elk, which is equivalent to k = 5, and thereafter, predicted the relevant binary CWD phenotypes.

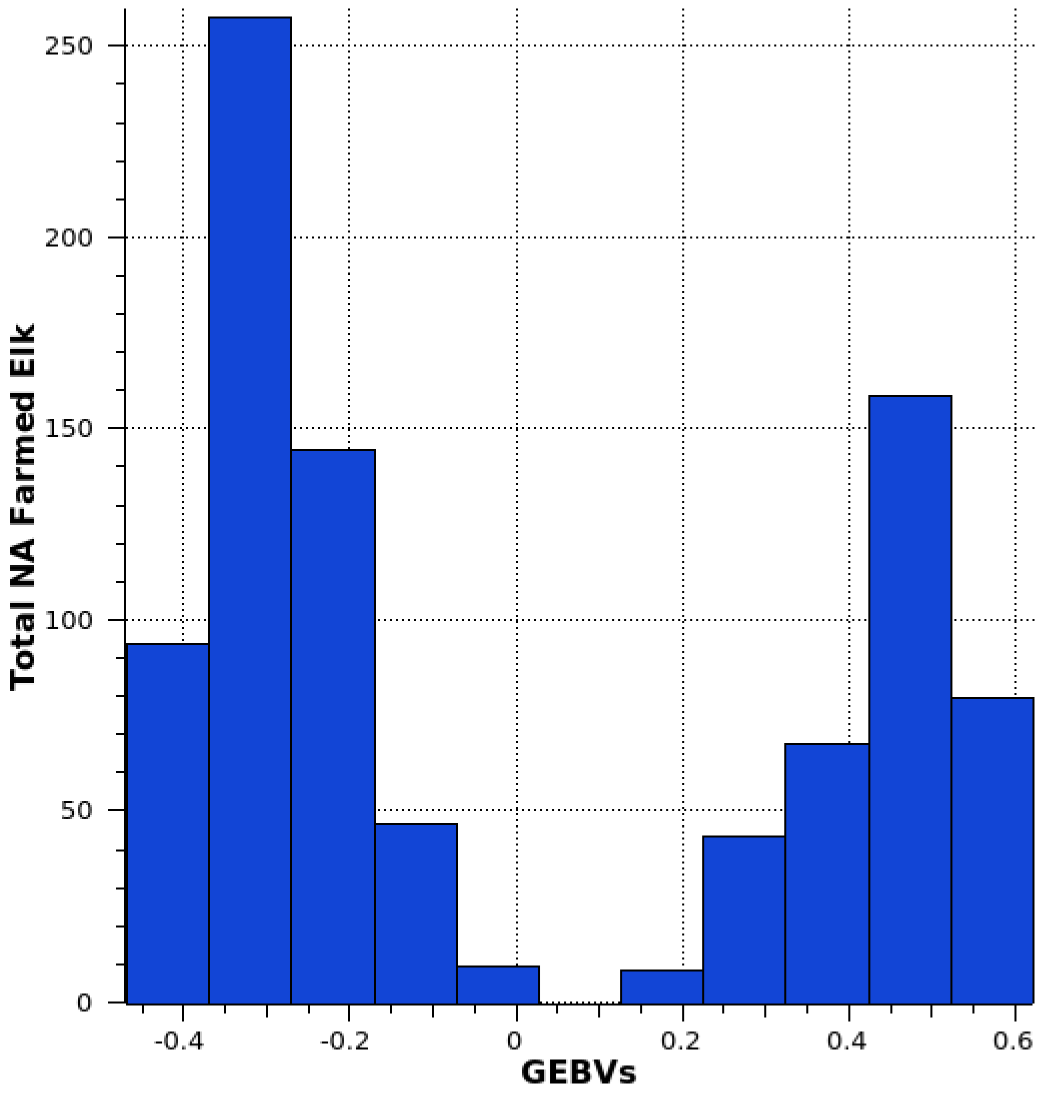

Figure S2 (A). Histogram of genomically estimated breeding values produced for the binary CWD trait using GBLUP with full dosage compensation and no fixed-effect covariates (n = 173,674 variants) for 904 farmed North American (NA) elk (*Cervus canadensis*).

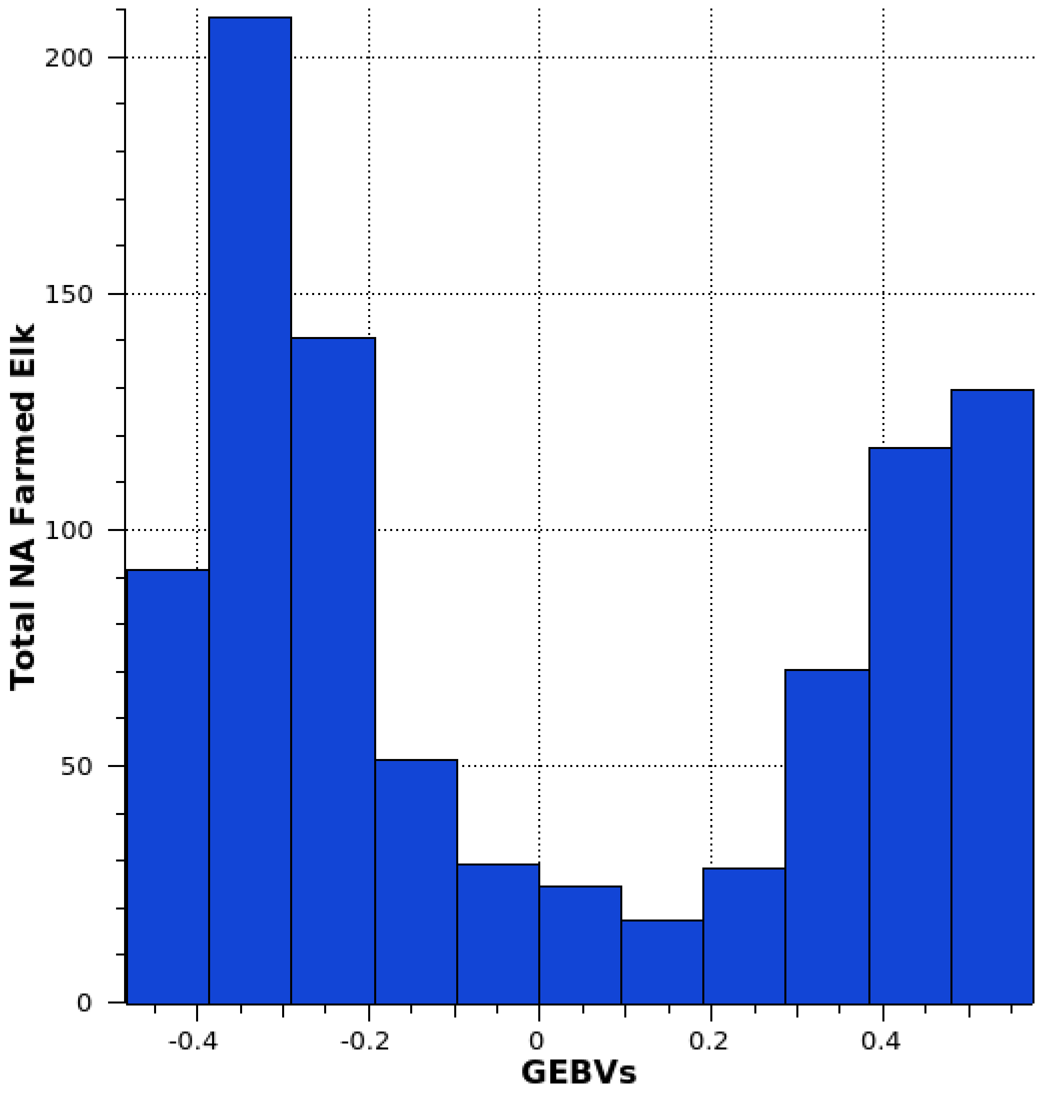

Figure S2 (B). Histogram of genomically estimated breeding values (GEBVs) produced for the binary CWD trait using GBLUP with full dosage compensation and no fixed-effect covariates (n = 173,674 variants) for 904 farmed North American (NA) elk (*Cervus canadensis*). For this analysis we randomly masked (i.e., set as missing) the known binary CWD diagnostic phenotypes for 181 farmed NA elk, which is equivalent to *k* = 5, and thereafter, predicted the relevant GEBVs.

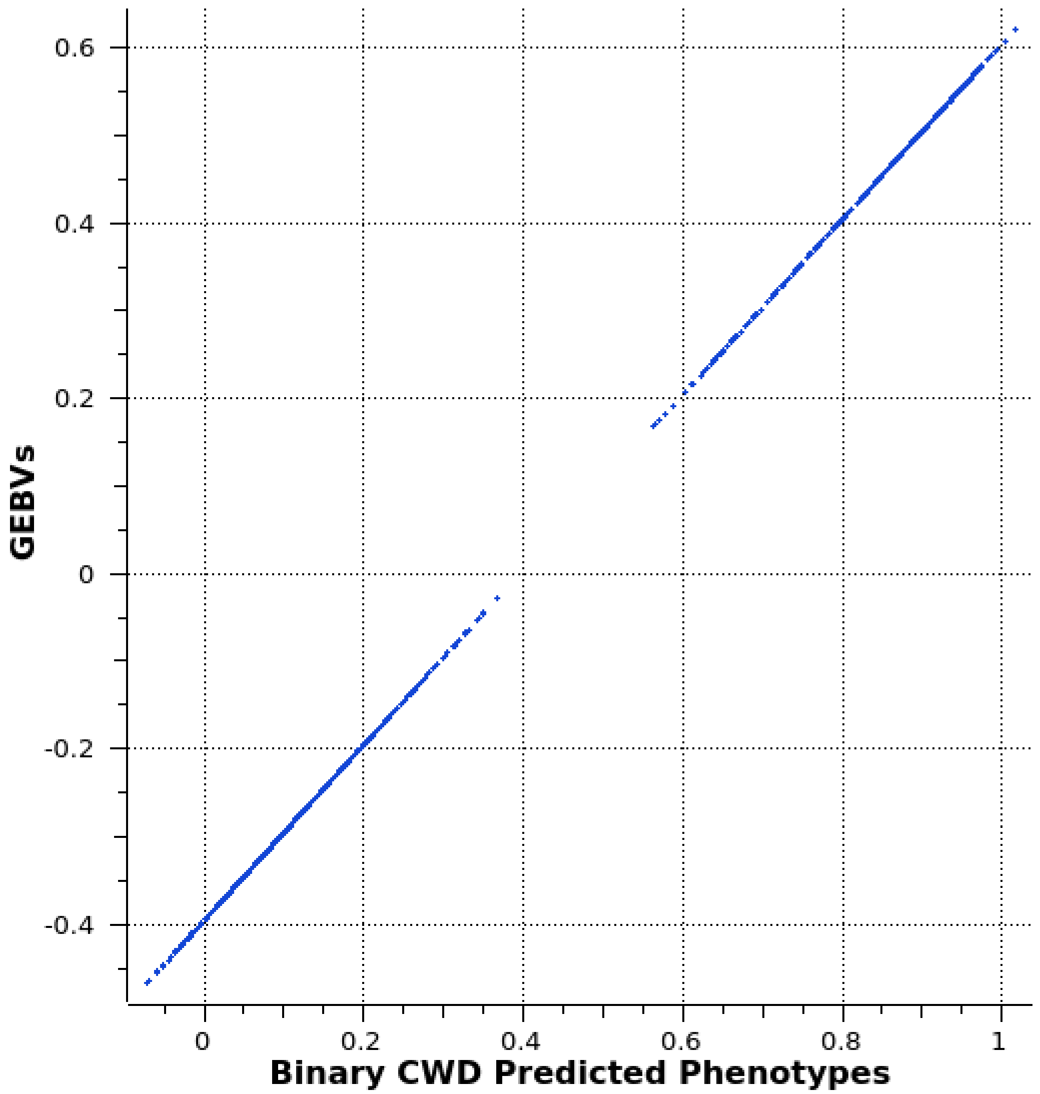

Figure S3 (A). Relationship between the genomically estimated breeding values (GEBVs) for the binary CWD trait and the predicted binary CWD phenotypes produced using GBLUP with full dosage compensation and no fixed-effect covariates (n = 173,674 variants) for 904 farmed North American (NA) elk (*Cervus canadensis*).

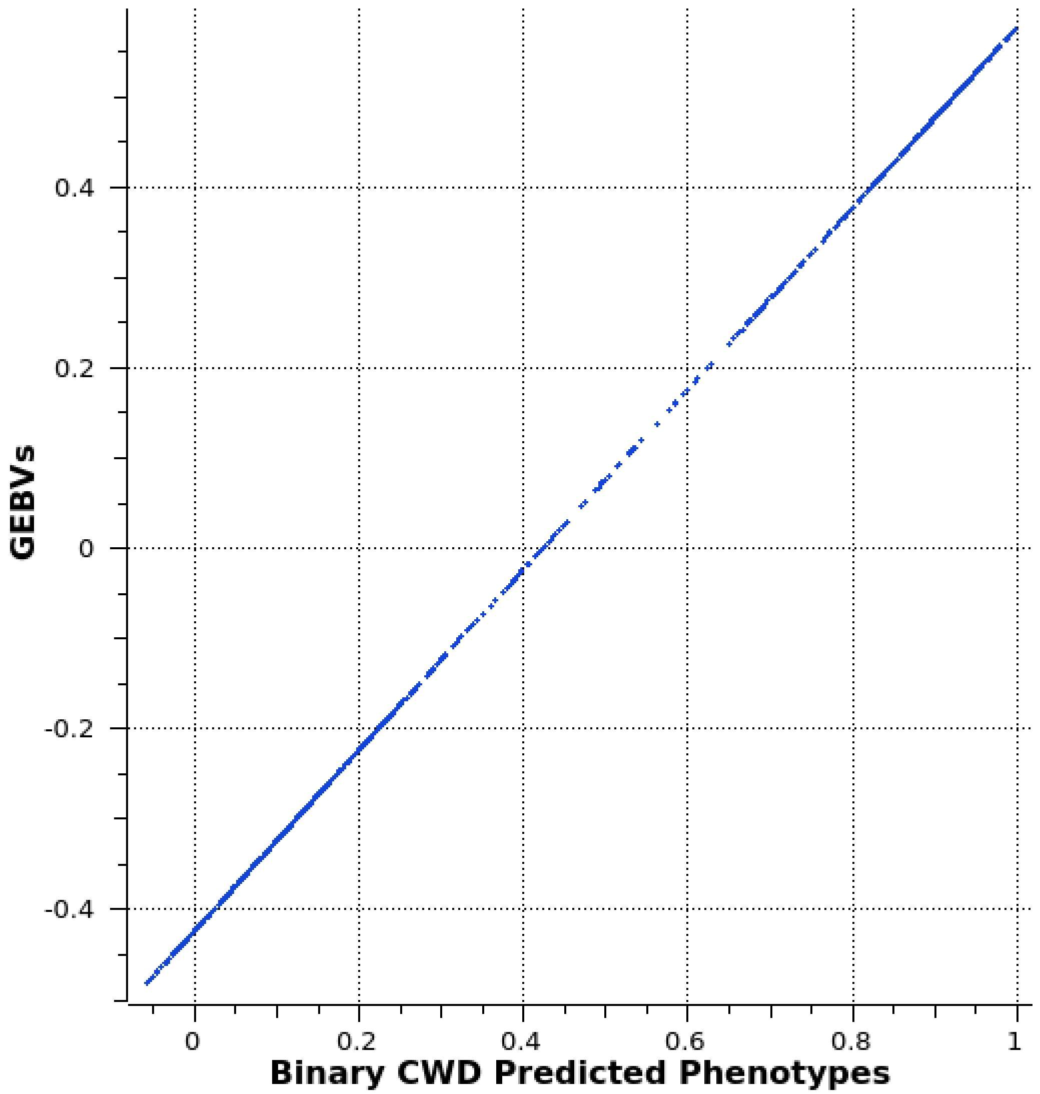

Figure S3 (B). Relationship between the genomically estimated breeding values (GEBVs) for the binary CWD trait and the predicted binary CWD phenotypes produced using GBLUP with full dosage compensation and no fixed-effect covariates (n = 173,674 variants) for 904 farmed North American (NA) elk (*Cervus canadensis*). For this analysis we randomly masked (i.e., set as missing) the known binary CWD diagnostic phenotypes for 181 NA farmed elk, which is equivalent to $k = 5$, and thereafter, predicted the binary CWD phenotypes and produced GEBVs for all 904 elk.
